# State of the Field in Multi-Omics Research: From Computational Needs to Data Mining and Sharing

**DOI:** 10.3389/fgene.2020.610798

**Published:** 2020-12-10

**Authors:** Michal Krassowski, Vivek Das, Sangram K. Sahu, Biswapriya B. Misra

**Affiliations:** ^1^Nuffield Department of Women’s & Reproductive Health, University of Oxford, Oxford, United Kingdom; ^2^Novo Nordisk Research Center Seattle, Inc, Seattle, WA, United States; ^3^Independent Researcher, Bengaluru, India; ^4^Independent Researcher, Namburu, India

**Keywords:** machine learning, benchmarking, FAIR, integrated omics, multi-omics, reproducibility, visualization, data heterogeneity

## Abstract

Multi-omics, variously called integrated omics, pan-omics, and trans-omics, aims to combine two or more omics data sets to aid in data analysis, visualization and interpretation to determine the mechanism of a biological process. Multi-omics efforts have taken center stage in biomedical research leading to the development of new insights into biological events and processes. However, the mushrooming of a myriad of tools, datasets, and approaches tends to inundate the literature and overwhelm researchers new to the field. The aims of this review are to provide an overview of the current state of the field, inform on available reliable resources, discuss the application of statistics and machine/deep learning in multi-omics analyses, discuss findable, accessible, interoperable, reusable (FAIR) research, and point to best practices in benchmarking. Thus, we provide guidance to interested users of the domain by addressing challenges of the underlying biology, giving an overview of the available toolset, addressing common pitfalls, and acknowledging current methods’ limitations. We conclude with practical advice and recommendations on software engineering and reproducibility practices to share a comprehensive awareness with new researchers in multi-omics for end-to-end workflow.

## Introduction

In the last decade, the application of different individual omic studies (e.g., genomics, epigenomics, transcriptomics, proteomics, metagenomics) that aimed at understanding a particular problem in human disease (Karczewski and Snyder, 2018), agriculture (Ichihashi et al., 2020), plant science (Liu et al., 2016), microbiology (Quinn et al., 2016), and the environment have been successful to a great extent. These studies generate a plethora of data, which, with careful integration under a suitable statistical and mathematical framework, can help to solve broader queries pertaining to basic and applied areas of biology.

More generally, performing multiple omics research often means having datasets with very different data modalities originating from varied assay types and increased dimensionality. In a multi-omics workflow (e.g., while profiling RNA, protein, or metabolites) the transcriptomics dataset, from RNA-seq efforts, can generate hundreds to thousands of transcripts (and the isoforms). In comparison, an individual researcher can only profile a few thousand proteins (and the proteoforms) or a few hundred identified metabolites (and features). Thus, the information burden from the transcriptome can easily overshadow the more actionable discoveries made from proteins or metabolites that are closer to the phenotype (Fiehn, 2002). This can add annotation bias and lead to enrichment of noise if robust integrative frameworks for data handling are not employed. Multi-omics aims to identify molecular markers associated with biological processes by revealing the regulatory units across diverse omics layers (e.g., obtained from DNA, RNA, proteins, metabolites, *etc*.). Multi-omics provides insights in understanding the mechanisms underlying biological processes and molecular functions, interactions and cellular fate, whether *in vivo* or *in vitro*, to reveal molecular phenotypes. Multi-omics can support discovery of predictive or prognostic biomarkers and/or potentially repurposed and novel drug targets in the era of precision medicine. Thus, the ultimate purpose of applied multi-omics is to increase the diagnostic yield for health, improve disease prognosis and produce improved agricultural outputs via robust understanding of genotype-to-phenotype relationship.

**FIGURE 1:**
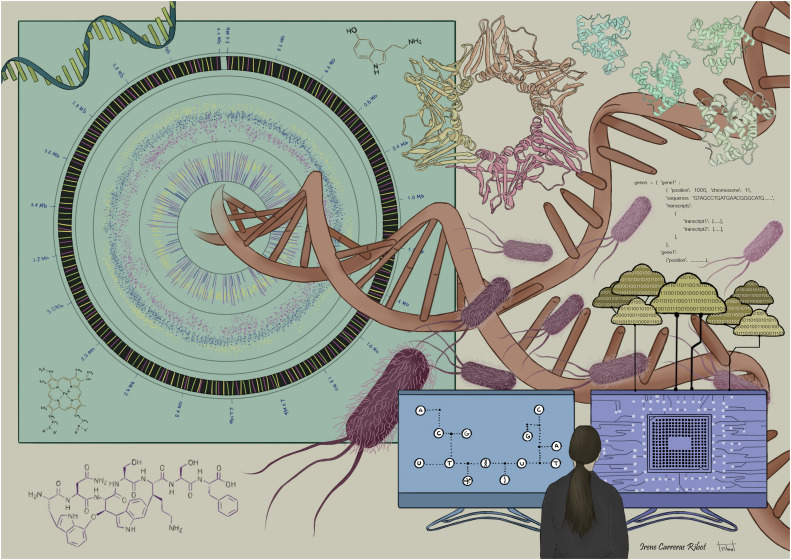
The complexity of multi-omics: merger of omics-driven biology, data science, informatics, statistics, and computational sciences.

Figure 1 represents an artist’s depiction of the complexity of multi-omics, a merger of omics-driven biology, data science, informatics and computational sciences. In spite of such challenges, the goal of multi-omics data is to support greater understanding of the overall biological process by bridging the gap of genotype-to-phenotype relationship.

We define multi-omics as three or more omic datasets coming from different layers of biological regulation – not necessarily within one level (exclusively derived from nucleic acid/DNA-derived, i.e., epigenomics, transcriptomics, and genomics). We have also not included proteogenomic that has immensely contributed to our improved understanding of protein sequences databases, gene annotations, gene models, and identification of peptides by interrogating genomics and transcriptomics while validating such protein data evidence using proteomics (Nesvizhskii, 2014). Further, this review does not discuss how other non-molecular data (i.e., phenotype data, clinical measures, imaging *etc*.) can be integrated with multiple omics datasets, as it entails a very different scope. While navigating this article, we recommend the readers consult Box 1, which contains the terms and concepts to support their understanding.

**BOX 1:** Terms, concepts, expressions, and definitions for clarity of readers foraying into multi-omics.

Terms, concepts, expressions	Definitions
Multi-omics/panomics/integromics/integrated omics polyomics/transomics cross-omics	An approach aiming to improve the understanding of systems regulatory biology, molecular central dogma and genotype-phenotype relationship by combining 3 or more different omics data.
Multi-table, Multi-block	Terms focusing on the format of the data rather than its nature, popular in chemoinformatics (among other fields); can (but does not have to) imply a larger number of features than observations in the integrated tables/blocks.
Multi-view	Method often used in the field of ML for learning heterogeneity in the data and identification of patterns. By comparison to multiple cameras viewing an object from different angles, in omics context, the object can vary – whether it’s “cell,” “organism,” or just “genome” viewed via different seq* techniques.
Multi-source	This term encompasses datasets that are derived from multiple sources of molecular assays. This terminology is used, for example by the joint and individual variation explained (JIVE) tool (O’Connell and Lock, 2016) during EDA.
Multi-modal	A term often used in omics in reference to multiple measurements methods done at molecular level to gain holistic insights of cellular machinery (e.g., one cell at a time). It is also popular in drug repositioning that involves integration of more nuanced *electronic health record* (EHR) data integration.
Central dogma of molecular biology	This is an explanation of the flow of genetic information within a biological system from DNA to RNA (transcription) to protein (translation) to metabolites (enzyme catalysis).
Machine learning (ML) method	Algorithm (a sequence of instructions) aimed at learning from data, with applications including exploration/dimensionality reduction (unsupervised methods, e.g., PCA, matrix factorization) and classification/prediction (supervised or semi-supervised methods)
Deep learning (DL) method	A subtype of ML using deep neural networks, composed of artificial neurons (signal aggregating or transforming units) arranged in layers; the depth of the DL refers to the number of “hidden” layers between the “input” (exclusive) and “output” layers (inclusive).
Fusion (Baldwin et al., 2020)	A specific type of integration that applies a uniform method in a scalable manner, to solve biological problems which the multi-omics measurements target.
Exploratory data analysis (EDA)	It is an approach that is heavily used in statistics, data science field during early data analysis steps often coupled with visualization.
Matrix factorization	A class of ML algorithms based on matrix decomposition, i.e., representation of a data matrix by two or more matrices (factors) that can be multiplied together to obtain the original matrix (or its approximation). It can be used for classification, prediction, or exploration.
Data heterogeneity	The data with a structural variation that can be explained by the composition of the analyzed dataset; encompasses both the clinical heterogeneity (e.g., presence of two groups with different genetic make-up due to ancestral differences, or different underlying etiologies of a disease) and technical heterogeneity (i.e., batch effects).
Meta-data	A table of organized information and instructions that helps to summarize the data properties in order to make it findable and usable for data analysis across same or multiple projects.
Git	A version-control system for tracking changes in source code and other documents during software development. Platforms such as Github and Gitlab are built on top of it.

## Why Is Multi-Omics Challenging?

Firstly, each individual omics analysis presents a multitude of challenges (Gomez-Cabrero et al., 2014; Misra et al., 2019). Multi-omics analysis inherits challenges from the single omics datasets, and confounds further analyses with other new challenges of the integration/fusion, clustering, visualization, and functional characterization (Pinu et al., 2019; Jamil et al., 2020). For instance, prior to integrating two or more omics, analysts or investigators can face challenges in terms of data harmonization (e.g., different data scaling, data normalization, and data transformation needs pertaining to individual omics dataset). Further, given dimensionality constraints posed while integrating large multiple omics data sets (e.g., a large population study with thousands of individual samples), the computational burden and storage space requirements can be limiting for a given study.

**FIGURE 2:**
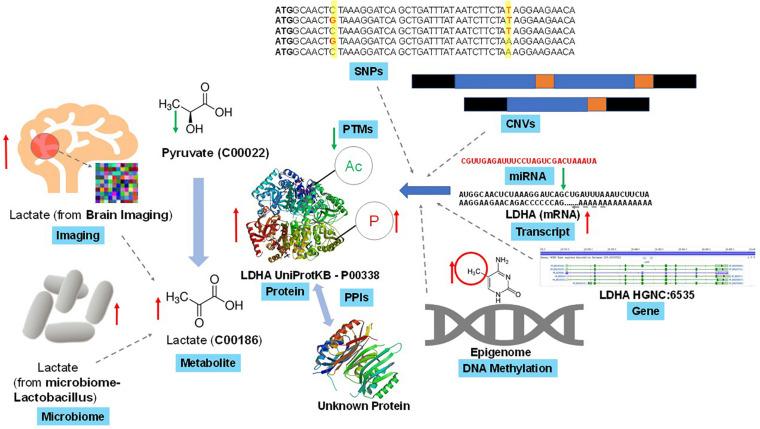
Example of complexity and interconnectivity of omics data sources in a multi-omics framework. A simple cellular endogenous metabolite, lactate is biosynthesized enzymatically from pyruvate (another metabolite) with the help of lactate dehydrogenase (LDHA, a catalytic protein). In turn this LDHA can interact with several known and unknown proteins through protein-protein interactions to regulate its own function, and itself is subjected to diverse post-translational modifications (PTMs) that regulate its catalytic function. Lactate measurement through techniques such as *in vivo* brain imaging in human or other model animals can generate lactate’s spatial distribution. Gut microbiome via Lactobacillus and other microbes can synthesize lactate and release into human physiological systems to contribute to lactate levels. Lactate biosynthesis regulation can be due to various levels of genetic (e.g., SNPs, CNV, etc.), transcriptomic, post-transcriptomic (e.g., miRNA) and/or epigenetics (e.g., DNA methylation) changes on the LDHA gene. Though this is one of the well-studied set of multi-omics interactions, but one can expect more complex and unknown interactions while integrating multi-omics datasets.

Even the identifiers (IDs) mapping – a prerequisite of some integration methods – is not an easy task when matching genes with associated transcripts or proteins (which is not a one-to-one correspondence), or a substantial challenge for other omics combinations, such as mapping genes to associated metabolites. Moreover, annotation of the omic entities (e.g., transcripts, proteins, and metabolites) with additional information, such as pathway membership and molecular characteristics, may require mapping IDs to various database systems (e.g., RefSeq or KEGG). Some of which may not cover all the omics of interest (e.g., metabolites are absent from RefSeq), while others may present outdated IDs due to delays after changes are made in the primary sources (e.g., KEGG GENE being based on RefSeq). The repertoire of identified and annotated molecules varies across omics, ranging from very good coverage of the genome, through a not-yet-complete picture of phosphoproteome and selective coverage of the metabolome. The challenges of metabolite identification may act as a bottleneck for advancement of the joint omics analyses. On the statistical side, unsupervised multi-omics methods can strengthen any signal, including systematic batch effect if present before quantitative measurements are taken, such as during sample acquisition, transport, processing logistics and operations. Failure to correct for such unwanted sources of technical variation, which may not be possible if the necessary information was not recorded during the sample handling steps, can misguide the overall integration process and impact the downstream interpretations and inferences (Kellman et al., 2020). Figure 2 exemplifies the complexity of individual omics data heterogeneity and data sources in the multi-omics framework in a human focused, biomedical study. In the section below, we identify three of the major challenges and pitfalls that explain the above scenarios:

### Data Wrangling

Also referred to as *“data munging,”* includes various levels of “transformation” and “mapping,” is critical to the multi-omics field. Transformation is accomplished by data scaling, normalization, and imputation that help harmonize different omics data together. Category of “mapping” can be the process of harmonization of IDs across various omics data types or simply annotating data across available meta-data, a labor-intensive process that requires massive one-to-one or one-to-many relationship operations. Careful registration of samples and robust metadata recording tables, with involvement of data generation and analysis teams can help circumvent this challenge and mitigate errors.

### Data Heterogeneity

Data heterogeneity is often another bottleneck while dealing with multi-omics data as these are generated via varied technologies (i.e., consider sequencing versus mass-spectrometry, or microarray versus mass-spectrometry scenarios) and platforms (i.e., targeted versus untargeted, high resolution versus single cell). Pre-processing steps pertaining to individual datasets may not help overall, especially when democratizing them under a unified framework still remains challenging. However, some tools have led to improved handling, such as similarity network fusion (SNF) (Wang et al., 2014), mixOmics (Rohart et al., 2017), Multi-Omics Factor Analysis (MOFA) (Argelaguet et al., 2018), among others. Their utility depends on matrix factorization, network fusion, canonical correlation, factor analysis, and are used for downstream feature extraction and feature selection purposes for phenotypic prediction. Efforts have focused on dimension reduction (Meng et al., 2016), integration approaches while running into multicollinearity (Meng et al., 2014), and integration issues when dealing with multi-omics and non-omics data (López de Maturana et al., 2019) as explained below.

### Dimension Reduction and Representation

Data representation, by means of dimensionality reduction that intends to project relationships of features (e.g., SNPs, transcripts, proteins, metabolites) across observations (e.g., samples, conditions, different omics layers) in a reduced space, is a common practice *a priori* in multi-omics efforts. Typically, following post-preprocessing after data normalization, data representation is applied to identify outliers, technical sources of variation – such as batch effects – and obvious biological patterns at each level of analysis – such as feature identification, extraction, and selection. This exercise aids in learning biological patterns and relationships of the data in bias identification and mitigation via appreciation of technical factors contributing to noise, adjusting them via batch effect correction, and identification of groups/sub-groups to confirm hypotheses of phenotypic conditions of interest in a given study. This is achieved by using clustering methods that are k-means, density-based, or graph-based, followed by generating visual representations using dimensionality reduction methods like principal component analysis (PCA), t-distributed stochastic neighbor embedding (t-SNE), and uniform manifold approximation and projection (UMAP) to capture linear and non-linear relationships in the data. However, this approach is often challenging given the complexity of the analytical space and the study goals due to latent patterns encoded in input samples originating from different omics layers, technologies and platforms. Such complexities in representation can be attributed to the lack of optimal tunable algorithms both at mathematical and statistical levels. These challenges are well documented in bulk gene expression studies that show that there is no single best latent dimensionality or compression algorithm for analyzing gene expression data (Way et al., 2020). Similarly, Hu and Greene (2018) proposed having a third-party evaluation by methods developers on unseen data while benchmarking autoencoder (unsupervised neural network) methods in single cell RNA-Seq (scRNA-seq) data for learning representations. These issues substantially change the results while interrogating high dimensional biological data. This problem is also applicable and extendable in multi-omics analytical space given the varied nature of data types in each omics layer with diverse biological modalities, such as while integrating single cell genome sequencing (genomics), RNA-Seq (transcriptomics), ATAC-Seq (epigenomics) and/or Bisulfite-Seq (epigenomics) together after pre-processing, batch-correction and normalization steps. Additionally, the data is also challenging to integrate as the relationship between multi-omics data layers can extend from one-to-one and one-to-many to many-to-many. This is also a very well-established concept in the Gene Regulatory Network (GRN) area of Systems Biology where gene-to-gene relationship establishments across various DNA, RNA, protein, metabolite, *etc*. are often better associated and represented using non-linear methods. Mutual Information (MI) based networks were found to perform better than other methods in such areas (Liu, 2017).

**FIGURE 3:**
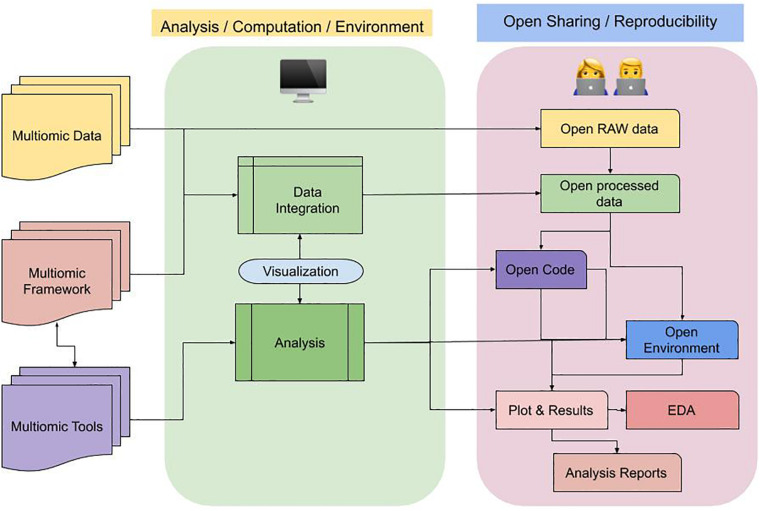
Flow diagram of best practice guideline in a multi-omics study for FAIR sharing. A multi-omic study entails data varied assays/sources/omics type, that can be integrated using various framework and tools. This process (represented in block with light-green) can be computationally intensive. As a by-product we get processed data, which can be taken forward to do multiple steps involving exploration, inferencing and interpretations. Sharing both the data and code alongside compute environment allows interoperability and non-reinventing the wheel. Here (represented in third block with light-purple), describes the open sharing of different components in a multi-omic project, the connected blocks that can eventually generate reproducible results in forms of reports for users.

In Figure 3, we demonstrate a flow diagram to adhere to best practice guidelines in a multi-omics study for FAIR data sharing.

## Before you Start: The Need for Consultation and Pilot Data Upfront

Only a robust study design can lead to error-free execution of a multi-omics workflow. Though there are several proposed study design considerations and guidelines available for individual omics in genomics (Honaas et al., 2016) and metabolomics (Chu et al., 2019), such comprehensive guidelines are not developed for multi-omic studies to our knowledge. It is not surprising that the study design guidelines for individual omics vary in scope and coverage since each omic field faces different challenges and opportunities. Without proper experimental design, poorly planned multi-omics efforts lead to analytical complexity, non-informative inferencing, exclusion of tangible interpretations, overriding true biological signals, and eventually feed into the reproducibility crises plaguing high throughput omics domains. Some of the considerations needed to overcome these issues include: (a) careful assessment of statistical power and effect size appropriate to the experimental design, (b) identification of confounders (e.g., sex, age, input materials) inherent to the data, biases (e.g., replicates: biological and technical) and sources of variations (e.g., batch, analytical, unwanted) that are anticipated in the course of data generation, (c) quality assurance (QA) and quality control (QC) measures that are associated with individual omics data generation and analytical platforms and (d) cross-validation measures implemented in cases of unavoidable biases.

### Sample Size and Statistical Power: Challenges and Opportunities

Different omics data require different numbers of samples to draw reliable conclusions. Reliability is dependent on false-discovery rate (FDR), which is influenced by the number of measured entities (i.e., transcripts/proteins/metabolites). Smaller omics data generation platforms such as microRNAs may need about 19 samples per experimental group to achieve a power of 0.8 at a fold change of 1.5 (Kok et al., 2018) with FDR < 0.1. Whereas, a set of 10,000 transcripts, each with at least 10 counts, would require a minimum of 35 samples per group for the same effect size at the same power and FDR control level, as calculated with *ssizeRNA* (Bi and Liu, 2016) using parameters pi0 = 0.8 disp = 0.1. The power calculation is not equally easy for each of the omics. While many tools were devised for transcriptomics/genomics power analysis, there are fewer dedicated tools available for metabolomics and proteomics studies. Only recently, a method to estimate an optimal sample size for multi-omic experiments was proposed (Tarazona et al., 2020) that addresses power calculation in multi-omics studies. This is one of the first comprehensive work that performed rigorous evaluations of relevant parameters across varied omic technologies (both sequencing and non-sequencing/i.e., mass-spectrometry based), built an open source tool (MultiPower^1^), that will enable future researchers to perform power and sample size estimation for their choice of multi-omics ED platforms while designing future studies and projects.

Further, pending cost-benefit tradeoff considerations, investigators typically decide on inclusion or exclusion of an individual omics experiment in a multi-omics setup. In certain cases, doubling the sample size is more informative than inclusion of an additional omics assay. For example, since small effects may not be clinically useful, increasing sample size may not be prudent when looking for biomarkers where assessing multi-omic panels may be more useful. When investigating disease subtypes, or patient stratification, a larger sample size may be desirable to achieve higher power in each of the subtypes. Subtyping of complex disease may benefit from diverse omics representation. Whereas, a study of biological mechanisms may benefit from related omics for a focused analysis of chosen omics types.

When planning a multi-omic analysis for a method that requires matched samples from all available omics datasets, the omic with the largest sample size requirement may dictate the need for such a large sample size across all analyzed omics. Here we provide two scenarios explaining the issue. For instance, in scenario one, when a study recruits 20 patients, collecting their biofluids for: genotyping, RNAseq, and metabolomics; and receives 19 genotypes, 18 transcriptomes and 17 metabolomes, one may incorrectly infer that the data is representative of 17 patients, but actually the failed samples (and QCs) originate from different patients across platforms. In reality, the experiment may result in only 14 patients with a complete set of measurements post QC across all omics. In scenario two, one study can recruit up to 100 patients but cannot afford to complete all three experiments on every patient. Hence, the researchers may decide to acquire data for 1000 genotypes, as those are affordable, and then split the transcriptome and metabolome equally to 70 a piece. This translates to matched samples for only 70 patients, thereby indicating missingness of data within and across omics layers. While the resulting missingness appears suboptimal, the integrative multi-omic design may allow researchers to decrease the sample size requirement; this is due to the increased potential of integrative analysis (Rappoport and Shamir, 2018). In this case, one can handle such sparsity by making a trade-off between genes (highly variable) given sample size is low or use sparsity methods in underlying available multi-omics frameworks. Moreover, the researchers may not consider each of the omics as equally important for their biological question and may be willing to focus on observations of larger effect sizes in an individual omics, which would drive up the cost of the project. One of the recent works on such parameter harmonization and power size estimate in the realms of multi-omics is very well captured and addressed elsewhere (Tarazona et al., 2020).

Sample size is also an important consideration for multi-omic studies of rare diseases or difficult-to-access tissue, such as cerebrospinal fluid or endometrial tissue. These studies may struggle to recruit larger numbers of patients, exacerbating the disproportion between the number of samples and features. The early integration multi-omics strategies may be a good fit for such low sample-size experiments, as those allow to detect more subtle effects if consistently present across analyzed omics (Rappoport and Shamir, 2018). When choosing whether to include an additional omics layer, we advise a thorough examination of previous studies combining the omics intended for use, as the cost/benefit trade-off while including an additional omic layer may vary (information gain), the omic characteristics (e.g., signal/noise ratio) and the availability of validated computational methods for specific omics type or in combination.

### Consulting Platform Experts and Incorporating Pilot Data

Given that the platform-specific characteristics–such as varying dispersion rates–require tailored solutions, researchers may require different parameters for RNAseq versus microarrays in transcriptomics, for liquid chromatography-mass spectrometry (LC-MS) versus aptamer-based proteomics or targeted versus untargeted metabolomics. Expert consultation is prudent before start of a pilot study to gauge the overall feasibility of the experiments and capabilities of the individual platforms in yielding optimal features (Tarazona et al., 2020), to design the final multi-omics study (note: the number of features or predictors in a given study is often denoted by ‘p’).

## Current State of the Art and the Tools

Multi-omics approaches can broadly be categorized as:

(a) Supervised – classification tasks that include discrete outcomes, such as disease/control status, and prediction tasks like that of continuous outcome, (e.g., survival, pain score).
(b) Exploratory – unsupervised clustering (e.g., disease subtype discovery) and relationship-based analysis (e.g., correlation/covariance and network models).

Even, over the past decade or so, a diverse array of multi-omics tools have been developed (Misra et al., 2019; Subramanian et al., 2020), some of which have gained popularity in recent years, including: mixOmics (Rohart et al., 2017), SNF (Wang et al., 2014), Paintomics (Hernández-de-Diego et al., 2018), 3Omics (Kuo et al., 2013), miodin (Ulfenborg, 2019), and MOFA (Argelaguet et al., 2018), as evident from the growing number of applications, user support requests, and citations. Table 1 presents types of tools and resources which are useful for execution of a multi-omics workflow, together with the examples for each of the categories.

**TABLE 1:** A complied list of various resources for supporting FAIR and interactive multi-omics study.

Serial No	Tools	Purpose	Link	References (if any)
**Popular/Emerging Multi-omics Tools**				
1	mixOmics	A tool with a framework that provides wide range of multivariate statistical methods for exploratory data analysis (EDA). This involves features identification, extraction and selection.	http://mixomics.org/	Rohart et al., 2017
2	MOFA	A probabilistic multi-omics factor analysis-based framework that involves EDA and data integration. (Unsupervised)	https://github.com/bioFAM/MOFA	Argelaguet et al., 2018
3	SNF	A multi-view network and fusion analysis framework for feature extraction, pairwise similarity, clustering, classification, etc.	https://cran.r-project.org/web/packages/SNFtool/index.html	Wang et al., 2014
4	miodin	A multi-level statistical framework involving vertical and horizontal integration of multi-omics data.	https://algoromics.gitlab.io/miodin/	Ulfenborg, 2019
5	Paintomics	A web-based systems biology tool for multi-omic integration and visualization across multi-species.	www.paintomics.org	Hernández-de-Diego et al., 2018
6	3Omics	A web-based application for integration and analysis of multi-omics data.	https://3omics.cmdm.tw/	Kuo et al., 2013
**Data Sharing**				
1	OmicsDI	An aggregated database facilitating the discovery of heterogenous published omics datasets across studies.	http://www.omicsdi.org	Perez-Riverol et al., 2017
2	Zenodo	A general-purpose open-access data, softwares, etc repository that allows user to obtain a citable DOI.	https://zenodo.org/	NA
3	OSF	An open platform to enable collaboration by registering research projects, materials, data and documentation.	https://osf.io/	NA
**Code Sharing**				
1	GitHub	A version-controlled code sharing and collaborative platform.	https://github.com/	NA
2	BitBucket		https://bitbucket.org/	NA
3	GitLab		https://about.gitlab.com/	NA
**Workflow Sharing**				
1	Common Workflow Language (CWL)	An open standard for describing analysis workflows which makes them portable and scalable across a variety of software and hardware environments.	https://www.commonwl.org/	Amstutz et al., 2016
2	Nextflow	An enterprise level workflow language for writing scalable and reproducible scientific pipelines.	https://www.nextflow.io/	Di Tommaso et al., 2017
3	Snakemake	A workflow language for writing scalable and reproducible scientific pipelines.	https://snakemake.readthedocs.io/en/stable/	Koster and Rahmann, 2012
**Environment Sharing**				
1	Conda	A package manager and computation environment management system.	https://docs.conda.io/en/latest/	NA
2	Bioconda	A channel for the conda package manager specializing in bioinformatics software.	https://bioconda.github.io/	Grüning et al., 2018
3	Docker	A container platform that provided OS-level virtualization for providing reproducible computation environment.	https://www.docker.com/	NA
4	BioContainers	A community-driven project that provides docker based containerized bioinformatics software.	https://biocontainers.pro/	da Veiga Leprevost et al., 2017
5	renv	A R-package that helps create reproducible environments for R-based projects.	https://rstudio.github.io/renv/	NA
**Serial No**	**Tools**	**Purpose**	**Link**	**References (if any)**
**Data Visualization**				
1	Shiny	A framework in R for doing GUI based interactive applications.	https://shiny.rstudio.com/	NA
2	Plotly	A cross language interactive plot library.	https://plotly.com/	NA
3	bokeh	A Python library for Interactive data visualization in browser.	https://bokeh.org/	NA
4	D3,js	A JavaScript library for producing dynamic, interactive data visualizations in web browsers.	https://d3js.org/	NA
5	Cytoscape	A platform for network data integration, analysis, and visualization.	https://cytoscape.org/	NA

## Advances and Limitations in Benchmarking

The increasing reliance on computational methods necessitates systematic evaluation (benchmarking) of the omics data analysis tools and methods (Mangul et al., 2019). The key challenges in omics-scale benchmarking of computational tools, include: acquisition of “gold standard” datasets (providing unbiased ground truth), incorporating new methods for establishing benchmarks as they are published (continuous/extendible benchmarks), and ensuring reproducibility in the context of increasing complexity of the software involved (Mangul et al., 2019; Weber et al., 2019; Marx, 2020). Each of these challenges is amplified in the multi-omics field – matched omics measurements are more difficult to obtain, novel methods can rely on specific combinations of omics being available (limiting opportunities for extending previous benchmarks) and software requirements may increase in complexity as authors strive to combine results of multiple state-of-the-art single-omics tools for improved multi-omics performance.

Gold standard datasets that incorporate multiple omics and provide unbiased ground truth are a prerequisite for proper systematic evaluation of multi-omics methods. The Cancer Genome Atlas (TCGA), which includes genomic, epigenomic, transcriptomic, proteomic, and clinical data for 32 cancers (Blum et al., 2018), is a landmark dataset for multi-omics methods development. Our literature search reveals that references to TCGA are enriched in the multi-omics computational method articles compared to other article types (48.5% versus 19.7%, OR = 3.83, *p*-value = 4.5 × 10^–07^, full-text analysis of the open-access PMC subset; see below for methods). While many other multi-omics datasets exist (e.g., for inflammatory bowel disease^2^ or amyotrophic lateral sclerosis^3^); the community is yet to decide on a suitable “gold standard” across varied disease and tissue types, other than cancers. This process will require the expertise of domain-experts and characterization of statistical and technical properties of the datasets (e.g., presence of batch effects, analysis of confounders) (Marx, 2020).

A handful of notable multi-omics benchmarks are available, comparing: multi-omics and multi-view clustering algorithms (Rappoport and Shamir, 2018), multi-omics dimensionality reduction (Cantini et al., 2020) and multi-omics survival prediction methods (Herrmann et al., 2020). All three benchmarks were performed using the TCGA cancer data. While it is beneficial to use the same dataset for comparison, results obtained this way cannot be generalized beyond cancer biology, nor applied to the integration of other omics – such as metabolomics, or microbiome data – that are not included in the TCGA. With new multi-omic tools being developed, a comprehensive comparison against existing tools is clearly missing, primarily attributable to limited availability of “gold standard” data sets. Other than the widely used multi-omics datasets from TCGA cancer patients, only limited studies incorporate simulated datasets, such as the R InterSIM package–which is also based on data dependence structure from the TCGA cancer studies.

Even the evaluation of a method on real-world data can be limited by the quality of the ground truth. One such scenario is the multiple multi-omic methods benchmarking against breast cancer subtypes that are primarily derived from a transcriptome based PAM50 signature (Bernard et al., 2009; Mathews et al., 2019). Such ground truth may favor the transcriptomic signal that could explain the limited perceived benefit of the multi-omics methods over single omics. Therefore, alternative strategies may be beneficial in the evaluation of subtypes derived by multi-omics methods (e.g., survival, drug response).

Given the limitations in the systematic characterization of multi-omic tools and methods, researchers need to choose tools that are either well benchmarked in appropriate scenarios and/or evidenced in multiple observational studies and systemically evaluated.

## *Fair*ification of Multi-Omics Efforts

Reproducing results in the multi-omics domain is understandably challenging because of the use of diverse data analysis methods, tools, and statistical processing, but as a research community we strive to make research efforts conform to findability, accessibility, interoperability, and reusability (FAIR) standards. Thus, the latest advancements in data sharing and environment replication can be leveraged to address this issue. In the following sections, we introduce means and approaches to share data, code, workflow, and environment while executing a multi-omics analysis to enhance the FAIRness (Wilkinson et al., 2016) which is suboptimal in the multi-omics field.

**FIGURE 4:**
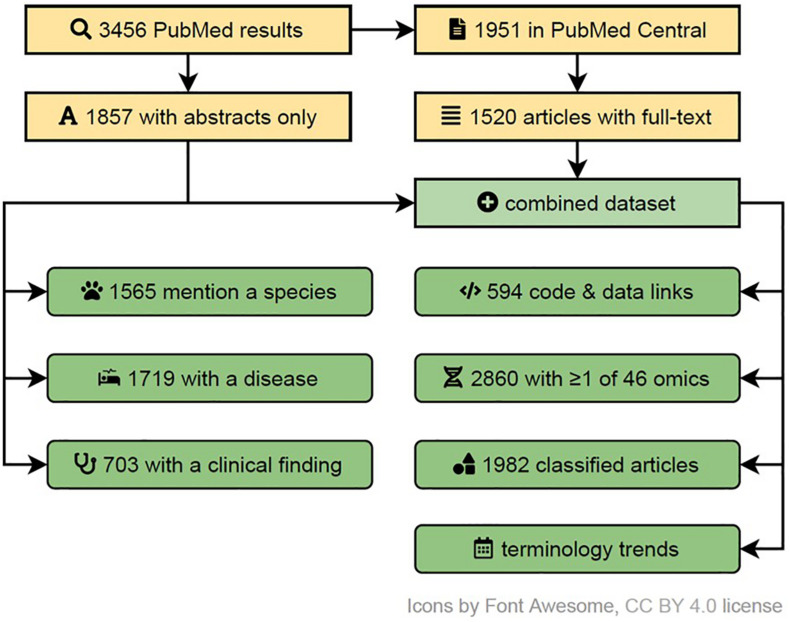
A systematic flow diagram to screen multi-omics literature in PubMed indexes articles (up to July 2020). This flow diagram represents the various steps of inclusion and exclusion criteria used to identify varied characteristics and attributes associated with published multi-omics studies. A detailed self-explanatory method with reproducible code are available at https://github.com/krassowski/multi-omics-state-of-the-field.

In order to determine the usage of multi-omics terms and their variants in the literature, to capture the trends in similar research domains, to identify their FAIRness in publications and the overrepresentation of research areas in them, we performed a systematic search (see Figure 4). We searched the PubMed database for articles pertaining to multi-omics on 25^th^ July 2020, using fourteen terms (multi| pan| trans| poly| cross-omics, multi-table| source| view| modal| block omics, integrative omics, integrated omics and integromics) including plural/singular and hyphenated/unhyphenated variants and their combinations. The search was automated via Entrez E-utilities API and restricted to Text Words to avoid matching articles based on the affiliation of authors to commercial entities with such names. Further, the full text and additional metadata were retrieved from the PubMed Central (PMC) database for the open access subset of articles. Feature extraction was performed via n-gram matching against ClinVar (diseases and clinical findings) and NCBI Taxonomy (species) databases, while omics references annotation was based on regular expressions capturing phrases with suffix “-ome” or “-omic” (accounting for multi-omic phrases and plural variants). All disease and species matches were manually filtered down to exclude false or irrelevant matches and to merge plural forms. The article type was collated from five sources: (a) MeSH Publication Type as provided by PubMed, (b) community-maintained list of multi-omics software packages and methods available at https://github.com/mikelove/awesome-multi-omics [accessed on *2020-06-24*], (c and d) PMC-derived: Article Type and Subjects (journal-specific) and (e) manual annotation of articles published in Bioinformatics (Oxford, United Kingdom), due to lack of methods subject annotations in PMC data for this journal. The details and code are available in the online repository: https://github.com/krassowski/multi-omics-state-of-the-field.

**FIGURE 5:**
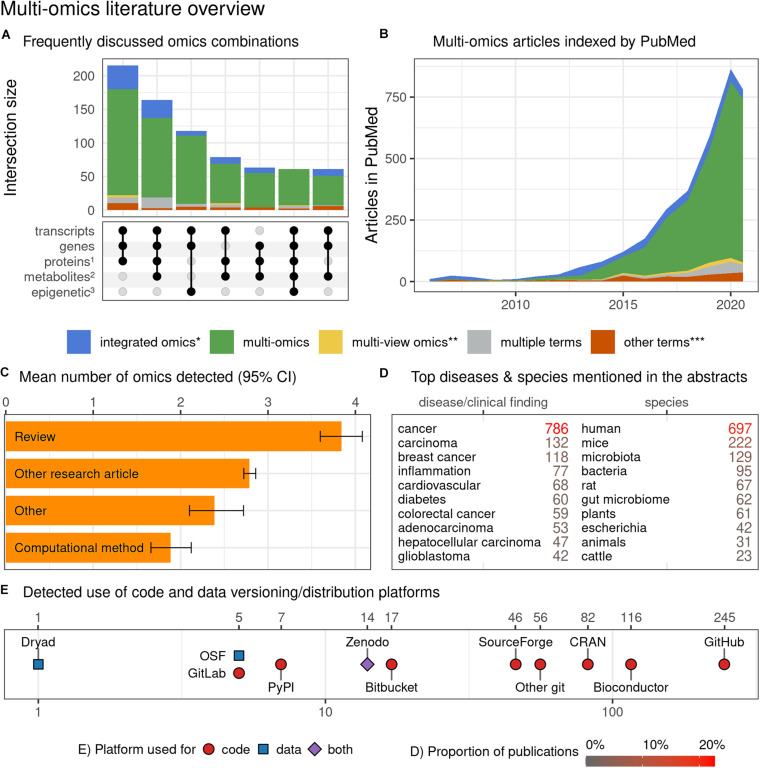
Characterization of multi-omics literature based on a systematic screen of PubMed indexed articles (up to July 2020). **(A)** Combinations of omics (grouped by the characterized entities) commonly discussed occurring together in multi-omics articles (intersections with ≥ 3 omics and at least 50 papers). *The proteins group (1) also includes peptides; the metabolites group (2) includes other endogenous molecules; the epigenetic group (3) encompasses all epigenetic modifications.* **(B)** Trend plot representing the rapidly increasing number of multi-omics articles indexed in PubMed (also after adjusting for the number of articles published in matched journals – data not shown); the dip in 2020 can be attributed to indexing delay which was not accounted for in the current plot. **(C)** Distribution of article categories that mention different numbers of omics; while it is understandable that multi-omics “Review” category discusses many omics, the “Computational method” category articles appear to lag all other article category types. The detected number of omics may underestimate the actual numbers (due to the automated search strategy) but should put a useful lower bound on the number of omics discussed. Bootstrapped 95% confidence intervals around the mean are presented with the whiskers**. (D)** The number of articles mentioning the most popular clinical findings, disease terms (here screening is based on ClinVar diseases list) and species (based upon NCBI Taxonomy database). Both databases were manually filtered down to remove ambiguous terms and merge plural/singular forms. Only the abstracts were screened here**. (E)** The detected references to code, data versioning, distribution platforms and systems (links to repositories with deposited code/data); both the abstracts and full-texts (open-access subset, 44% of all articles) were screened. No manual curation to classify intent of the link inclusion (i.e., to share authors’ code/data vs. to report the use of a dataset/tool) was undertaken. The details of the methods with reproducible code are available at github.com/krassowski/multi-omics-state-of-the-field. The comprehensive search terms (see the online repository for details) were collapsed into four categories; integrated omics (*) includes integromics and integrative omics, multi-view (**) includes multi-view| block| source| modal omics, other terms (***) include pan-, trans-, poly-, cross-omics.

The results of this systematic literature screen led to various interesting conclusions, as shown in Figures 5A–E. Primarily, our analysis revealed that multi-omic studies tend to focus on three layers of omics encompassing transcripts, genes, and proteins. This is followed by omics layers including metabolites and epigenetic modifications and combinations thereof (Figure 5A). A search of PubMed articles revealed that “multi-omics,” as a terminology, is dominant over “integrated omics” and other omics-associated terms with an incremental trend since 2010 (Figure 5B). The search for “-ome” and “-omic” terms suggested that review articles tend to discuss the highest number of distinct omics, while computational methods articles appear to discuss the fewest, suggesting a potential disparity between the abilities of available computational tools and the ambitions and needs of the multi-omics community (Figure 5C). Of the disease terms, the multi-omic studies most frequently featured “cancer” and “carcinoma,” while among the searched species “human” and “mice” dominated, indicating little representation of non-model species, organisms and biological systems. Articles mentioning “cancer” in title or abstract were overrepresented among the multi-omic articles when compared to other articles from the same time span, from the same journals and weighted by journal frequency in the multi-omics subset (22.7% vs. 7.5%, OR = 3.04, *p* < 10^–104^) (Figure 5D). Toward FAIR sharing of data and code, “GitHub” appears to be the most popular platform, followed by “Bioconductor” and “Comprehensive *R* Archive Network (CRAN),” among many others (Figure 5E). Below we share few topics contributing to FAIR approaches:

### Data Sharing

Different public databases are in place aiming to store and share specific kinds of omics data types as public repositories [e.g., genomics data in NCBI-SRA (Leinonen et al., 2011), GEO (Barrett et al., 2012) and EBI-ENA (European Bioinformatics Institute, 2016), proteomics data at PRIDE (Vizcaíno et al., 2016) and ProteomeXchange (Vizcaíno et al., 2014), or metabolomics data at MetaboLights (Haug et al., 2013), Metabolomics Workbench (Sud et al., 2016) and GNPS-MASSIVE (Wang et al., 2016)]. Only recently, have there been efforts to link these databases in a discoverable manner in the form of OmicsDI (Perez-Riverol et al., 2017). Mostly, raw sequences or very specific processed (count tables) data are being submitted to those databases, whereas, the intermediate outputs and analysis files are not shared, thus preventing reproducibility. The following resources can alleviate such scenarios: (a) Zenodo: allows users to upload raw data files, tables, figures and code. It supports code repositories, with GitHub integration, in addition to providing digital object identifiers (DOIs), and (b) OSF (Open Science Framework) (Foster and Deardorff, 2017): provides users with a platform where projects can be hosted with varied data types and file formats and contains a built in version control system. It also supports DOIs while promoting open source sharing that adheres with the FAIR guidelines.

However, adoption of such resources appears low in the multi-omics field as evident in our meta-analysis, with only 0.58% of publications (20 out of 3455 screened) linking to Dryad, OSF or Zenodo (Figure 5E).

### Code Sharing

To enable FAIR sharing of code, a data analyst can explore one of the multiple venues available that publicly hosts codebases. These are: (a) GitHub, (b) Bitbucket, and (c) GitLab. All of these platforms use the Git system to provide version control. Also, native Markdown and Jupyter based notebooks render support for providing an exploratory data analysis (EDA) narrative alongside code and its output.

### Workflow Sharing

As multi-omic analyses are often multi-step with each output being the input of another, in order to increase the efficiency workflows can be written with Domain Specific Languages (DSL) such as: (a) Common Workflow Language (CWL) (Amstutz et al., 2016), (b) Nextflow (Di Tommaso et al., 2017), (c) Snakemake (Koster and Rahmann, 2012), and (d) Galaxy-workflows (McGowan et al., 2020).

### Environment Sharing

The entire data analysis environment can be created and shared, saving time and aiding reproducibility (i.e., version control). Even accessing the intrinsic versioning information of each tool helps users in terms of interoperability, however, command line version handling parameters (e.g., −v/−V) are sometimes missing. The correction to a multi-omics clustering methods benchmark highlights the need for specifying the computational environment down to the processor architecture details (32 or 64 bit) (Rappoport and Shamir, 2019). As investigators attempt to build upon state-of-the-art implementations from various domains, like machine learning (ML), genetics, cell biology, the dependency on tools using different programming languages is incremental and some require a dedicated runtime environment (e.g., R and/or Python). Dedicated tools can help researchers who try to combine packages written in different languages in a single analysis workflow by allowing transparent data exchange and the use of interoperable functions across languages. One example of such a tool is the Python-R interface *rpy2* (rpy2, 2020), which found use in recent multi-omics tools (e.g., ReactomeGSA, Griss et al., 2020) and research scripts (Neyton et al., 2019). However, the use of multiple complex runtime environments can result in (version) conflicts if versions are not properly matched. This hinders the reuse of proposed tools and reproduction of published results. For example, each version of rpy2 requires a specific version of Python and R. The problem is not limited to Python → R workflow – the complimentary R → Python interface, *reticulate* (Reticulate, 2020) can be challenging to configure.

In order to ease the burden of interoperability and reproducibility that investigators often face while analyzing large multi-omics datasets with available algorithmic packages, several environment sharing avenues can be implemented, for example: (a) Conda (Conda, 2020): a cross-language tool repository and environment management system. With a shareable configuration (in yml format) file, an entire analysis environment can be re-installed in another system. Bioconda (Grüning et al., 2018) is a conda based project specifically designed for bioinformatic tools. (b) Docker (Docker, 2020): a ready to use lightweight portable virtual container, where an environment can be established, with all the required tools, for a particular analysis and shared. Specifically, bioinformatics tools such as Biocontainer (da Veiga Leprevost et al., 2017) are available. (c) Packrat (Packrat, 2020) (recently superseded by *renv*) and checkpoint: dependency management packages specific to R, which help to create isolated and portable R environments. *Checkpoint* facilitated one of the previous multi-omics benchmarking efforts (Herrmann et al., 2020).

### Computational Power

Multi-omics analysis does not necessarily require high-performance computational resources, unless performing large scale consortia data extraction, transformation, load (ETL) tasks across a few hundred-thousand samples. However, some recent supervised multi-omics methods and packages can be computationally expensive given the amount of training that happens during the feature level analysis (e.g., Data Integration Analysis for Biomarker discovery using Latent Components (DIABLO), MOFA, *etc*.). Such bottlenecks can be overcome using a higher end central processing unit (CPU), high-performance computing cluster (HPC) and/or a cloud resource. The requirement of storing large downloaded files can be overcome using raw data streaming feature, however only a few tools support such feature.

### Regulatory and Ethical, Legal, and Social Implications (ELSI) Issues

Additionally, multi-omics allows researchers to make more inferences on individuals in the event of a security incident, and labs/clinics that do translational research are often under regulatory compliances that restrict any data upload to any server for analysis when patient information is involved. There are multiple regulatory compliance-related restrictions spanning data security, ethical, personal information *etc*., that can serve as bottleneck challenges. Alternatively, any researcher who develops a multi-omics tool for the community and makes it server/web/cloud-based should consider the needs of healthcare researchers who will often encounter restrictions when uploading such a dataset due to privacy concerns and other regulatory checks. In such cases, researchers can explore and take resources from non-open source enterprise level analytics platforms that can be either cloud-based or stand-alone if such enterprise platforms are Good Manufacturing Practices (GMP) certified, adhering to Health Insurance Portability and Accountability Act (HIPAA) and General Data Protection Regulation (GDPR). There can be additional regulatory compliances, given the data is produced by Clinical Laboratory Improvement Amendments (CLIA) certified entities. If all such regulatory compliances are in place, then patient data can be used in either a stand-alone third-party platform or uploaded in a web/cloud-based server for any analytics followed by inferencing under strict vigilance. For example, some commercial companies that have such cloud-based solutions include Amazon AWS, Google Cloud and MS Genomics (Microsoft Genomics, 2020). All of these platforms, together with other commercially available enterprise platforms like KNIME (KNIME4Bio | KNIME, 2020), can provide the necessary toolbox for multi-omics research and development.

## Application of Machine and Deep Learning (ML/DL) in Multi-Omics

Over the years, machine learning (ML) and/or deep Learning (DL) have become increasingly popular in biomedical research due to their ability to perform unsupervised and supervised analyses using large datasets to provide logical or probabilistic inference. In the current data-driven era, apart from the large text mining exercises, pattern recognition and medical imaging, ML/DL growth has contributed to analysis of large-scale high-dimensional data that are typically generated using high throughput omics assays. Their use and challenges in the multi-omics field are very well summarized in a recent review by Mirza et al. (2019) that discusses topics of integrative analysis encompassing dimensionality reduction/representation, data heterogeneity, data missingness, class imbalance and scalability issues. Other impressive applications of ML/DL are often encountered in regulatory genomics to study DNA-protein interactions and relationships. Some examples of relevant studies and models related to regulatory genomics approaches are available under http://kipoi.org/. However, much of ML/DL newer bioinformatics applications are developed in varied forms of supervised and unsupervised manner, such as specific neural networks models have been built for feature identification, extraction, and selection purposes. Some of these approaches in the DL space are discussed in the review by Ching et al. (2018). Such DL models have been extensively used for multi-omics integration purposes to predict better molecular signatures associated with improved patient survival and capture intricate relationship patterns for better clustering over conventional methods and drug response prediction. Such pattern extraction, selection and representation are often difficult to achieve solely by traditional linear modeling unless coupled with advanced non-linear models. Some methods and tools from the multivariate statistics/ML/DL area that have been developed for multi-omics integration include: (a) Multi-Omics Model and Analytics (MOMA), (b) Multiple Kernel Learning (MKL) (Wilson et al., 2019), (c) DIABLO (Singh et al., 2019), (d) a multi-omics late integration method (MOLI) (Sharifi-Noghabi et al., 2019), (e) multi-omics deep learning method (DCAP) (Chai et al., 2019), and (f) Multi-omics Autoencoder Integration (MAUI) (Ronen et al., 2019). Partly this can be attributed to the reasons described above and partly as described in the following paragraph.

Often simple models do not account for the principles of dynamics and kinetics that underlie a set of biological processes. Considering central dogma as the key hypothesis (Reinagel and Speth, 2016) of molecular life, for the entire process from replication through transcription to translation machineries that are at play, each of these biological processes (i.e., a disease) have pre and post events that are building more complex functions at each step adding up to the biological stochasticity. These stochastic events are often not well accounted for in simpler models as researchers tend to overgeneralize using mathematical modeling, calculus and/or statistics. Frequently, such strategies are not adopted in multi-omics experimental design and also, as datasets are not always longitudinal in nature, they can often lead to biases or ineffective generalization or approximation in multi-omics results. Another argument occurs when DNA and RNA are assumed as distinct genetic materials. DNA and RNA can work individually to bring about structural or functional protein consequences that lead to a phenotypic change. This was addressed to an extent by Koonin (2012), where central dogma is challenged by “*genetic assimilation of prion-dependent phenotypic heredity*,” and only a few phenotypes might fall under such categories and phenomena. This can be due to (a) genetic insults, like chromosomal instability and loss of function mutations that directly impact the translational process, (b) insults to RNA machinery without upstream DNA impact, while any abnormalities in the RNA phase impinges the translational events and (c) insults possibly seen in few systemic diseases where not everything is reliant on DNA or germline mutations, but rather due to abnormality in the underlying regulatory machineries during transcription or pre-translation stages. Such events can often be guided by upstream epigenetic insults like DNA methylation, histone modifications or even specific enhancer binding processes on a different gene promoter thus impacting overall transcription and translation, leading to a phenotype.

Even at the level of proteins, the regulome is often guided by protein-protein interactions, and those by kinases and phosphatases, are barely predictable from the genome. Similarly, regulations of metabolite levels (catabolic and anabolic processes leading to their levels in a given system) are not predictable from the enzyme levels, let alone their protein or DNA sequences. These kinds of upstream processes are often not well captured via omics technology, as our current models or frameworks are yet to be fully optimized and cannot generalize at such a level of non-linear system dynamic relationships that leads to specific phenotypic processes (Reinagel and Speth, 2016). Taken together, all of the above lead to the motivation of developing more advanced variants of ML/DL-based tools in biomedical research for multi-omics integration to improve understanding of genotype to phenotype relationships. However, these methods can be very computationally expensive and not robustly validated as they will be under continuous development.

## Data Visualization Tools

Visual representation is one of the most important ways of deriving interpretations and inferences with data in multi-omics. With the advent of high-dimensional data generation platforms, such as NGS technology and mass spectrometry, such representation has become very popular. Currently, there is a trend of developing dynamic web-based and stand-alone applications among the larger research community in diverse omics domains. These are often published alongside code for reproducibility of the results as an additional resource for other users in the research community to explore and for hypothesis development. Visualization avenues of multidimensional data in an interactive platform adheres to FAIR standards. The need for joint visualization of multiple omics datasets prompted the adoption of dashboarding applications, such as BioTools (Biotools, 2020) and WilsON (WIlsON, 2020). Dashboards display together multiple interactive panels with high-dimensional data and are available for the majority of data-exploration ecosystems (e.g., R, Python, Jupyter, Tableau). The interactive visualization tools and dashboards can be installed locally as stand-alone tools (e.g., in workstation/server) or can be completely web-browser based (e.g., launched locally from a server or a cloud-based platform).

Some of these popular tools that have found application in multi-omics are: (a) R-based Shiny (Shiny, 2020) apps. Numerous Shiny based apps help with exploratory data analysis for testing of hypotheses, given the end-user is able to grasp the underlying statistical models/frameworks that perform a required task of a specialized biological query. Such shiny apps (Dwivedi and Kowalski, 2018; Kmezhoud/BioCancer, 2020; WIlsON, 2020) can be launched both locally on a computer, server or even hosted publicly catering to a larger community of researchers. Binder (Jupyter et al., 2018) allows researchers to quickly create the computational environment needed to interact with research code and data shared online. *Voilà* (Voilà, 2020) turns Jupyter notebooks into standalone web applications. (b) Plotly (Plotly, 2020) (multiple languages; both open source and commercial) includes several tools designed for using these resources either in a stand-alone manner or in conjunction with other available frameworks (Zeng et al., 2019). In a way similar to Shiny, it supports creation of complex dashboards when used with Python-oriented Plotly Dash. (c) Python-based tools with or without integration servers like bokeh (Bokeh, 2020) enables Python users to create interactive web-based applications for end-users with front-end. (d) Network and other advanced visualizations, including JavaScript-based libraries such as D3.js (data-driven documents) (D3.js, 2020), have functionality amenable for web-based network tools creation. Cytoscape (Otasek et al., 2019; Cytoscape, 2020), available both as a JavaScript library for online visualizations (Cytoscape.js) and stand-alone application for EDA, is a popular tool employed in the field of systems biology. Bacnet (BACnet Stack, 2020) is another available framework for developing custom multi-omics analysis websites including network and other advanced visualizations.

## Computational Resources Needed for Multi-Omics Analysis

In the following sections, we provide pointers for using computational resources and expertise needed for executing a multi-omics experiment.

### Knowledge of Programming Languages and Frameworks

Provided below are a few programming languages that are relevant and applicable to experiments in multi-omics: (a) Bash scripting and Python are useful for basic data pre-processing and workflow organization, (b) C/C++/Java may be useful for development of performant methods and algorithms, (c) R and Python are *de facto* standard for statistical programming and data visualization in the omics context and (d) Shiny/Bokeh are visualization frameworks convenient for creating web-based interactive multi-omics functions.

### Computational Infrastructure

We advise learning to handle a standard Linux distribution, enterprise-level or open-source cloud-based computational interface, such as Google Colab in order to run workflows/pipelines for EDA and launching softwares/tools for performing any integrative multi-omics/bioinformatics related tasks. These infrastructures can feed into varied analytical tasks, such as data wrangling, data integration, data analytics, data visualization, and functional analysis. Given such varied data intensive tasks are associated with multi-omics analysis, more often users need resources for stand-alone workstations with well powered Central Processing Unit (CPU), servers having Graphical Processing Unit (GPU) or high-end computing infrastructures with Tensor Processing Unit (TPU). The need of a GPU or TPU is however needed while running end-to-end ML/DL models with high-volume features and parameters.

### Databases, Visualizers and Portals

Numerous portals, databases, and data-centric tools can be used for integrative multi-omics explorations. Examples of those are cBioportal (Gao et al., 2013) (Cancer Bioportal); Xena browser (Goldman et al., 2019) (UCSC Xena Browser is an online exploratory tool for analyzing public and private, multi-omic and clinical/phenotype datasets); ICGC Data portal (Zhang et al., 2011) (International Cancer Genome Consortium Data portal); ENCODE Data Portal (Davis et al., 2018) [The Encyclopedia of DNA Elements (ENCODE) is a public research project which aims to identify functional elements in the human genome]; FANTOM5 (functional annotation of the mammalian genome 5) (The FANTOM Consortium and the RIKEN PMI and CLST (DGT) et al., 2014) and The Human Protein Atlas (HPA) (Thul et al., 2017). It is also important to gain basic knowledge of the underlying methods employed in these large databases by reading the associated manuscripts, frequently asked questions and tutorials/vignettes in order to gain substantial knowledge before using them for exploratory purposes.

## Future Prospects and Conclusion

Challenges abound – from dealing with biological complexity, to over-simplified models, to technological limitations associated with data generation, to organization of high throughput data for comprehensible visualization, to drawing meaningful conclusions. In this treatise, we did not cover the success achieved with multi-omics in various domains of microbial, plant, animal, and biomedical research in recent times to keep the scope focused and relevant to a diverse audience.

In this document, we have not touched upon several upcoming and exciting areas of multi-omics research as they are yet to mature. For instance, single-cell multi-omics are currently driven with efforts mostly at the genomic (single cell DNASeq), transcriptional (e.g., single cell/single nuclear) and epigenomic (single ATAC-Seq, single cell bisulfite sequencing) levels. They are currently in the early stages of inception and, as more promising works will ensue, researchers will reach precision with efficient capture of single cell proteomics and metabolomics. Currently, some early single cell proteomics work is emerging in the mass spectrometry driven omics area of proteomics (e.g., SCOPE2) (Specht et al., 2019). Prevailing challenges remain in terms of maximizing information from a single cell (Macaulay et al., 2017) using current proteomics and metabolomics strategies, where barely a handful of metabolites are captured (Nemes et al., 2012). However, there are already some early exciting works of single cell multi-omics integration methods available that are upcoming in manifold [e.g., MAGAN (Amodio and Krishnaswamy, 2018), UnionCom (Cao et al., 2020) and non-manifold – such as LIGER (Welch et al., 2019) and MOFA+ (Argelaguet et al., 2020)]. Hopefully, these will be addressed and covered in future multi-omics efforts.

From collective experience and evidence, the key to effective exploratory data analysis, hypothesis generation and interpretations is reliant – to an extent – on understanding the underlying methods used to build or digest them and draw inferences. With more high dimensional biological data generation in various arms of biology, be it plant, microbial, developmental/disease biology, and future implementation of various multi-modal multi-omics, it will be more likely to observe growth of such ML/DL methods. Hence, the applied ML/DL community in the bioinformatics domain will have to generate models that are interoperable, stable, and well benchmarked at various regularizations (tunable) for users to derive robust reproducible results. Alternatively, such ML/DL developers and researchers can also clarify the uncertainty bounds associated with their tools for the user community. As a nascent field, there is a dearth of studies or benchmark tools and resources to direct an upcoming community, but this review serves as a guideline for future multi-omics researchers from a computational standpoint.

## Author Contributions

BBM and VD conceived the idea. MK performed the meta-analysis. MK, VD, SS, and BBM wrote the manuscript. MK and SS generated the tables and figures. All the authors have read, agreed to the content, and approved the submitted version of the manuscript.

## Conflict of Interest

VD currently works as a Post-Doctoral Researcher in Novo Nordisk Research Center Seattle, Inc. He did not receive any funding for this work. BBM works as a Computational Biologist in Enveda Therapeutics and did not receive any funding for this work. SS has no conflicts of interest. MK has no financial conflicts of interest, but he contributed to two of the discussed projects: rpy2 and Jupyter.

## Footnotes

1 https://github.com/ConesaLab/MultiPower/

2 https://ibdmdb.org/

3 http://data.answerals.org/

## Abbreviations

AI: artificial intelligence
API: application programming interface
DL: deep learning
EDA: exploratory data analysis
FAIR: findable, accessible, interoperable, and reproducible
FDR: false discovery rate
GPU: graphics processing unit
KEGG: Kyoto Encyclopedia of Genes and Genomes
ML: machine learning
MOFA: multi-omics factor analysis
NGS: next generation sequencing
OR: odds ratio
PCA: principal component analysis
PMC: PubMed Central
QC: quality control
R: statistical programming language R
SNF: similarity network fusion
TCGA: The Cancer Genome Atlas.
